# Collectanea Medica, Consisting of Anecdotes, Facts, Extracts, Illustrations, Queries, Suggestions, &c.

**Published:** 1819-06

**Authors:** 


### COLLECTANEA MEDICA,

CONSISTING OF

### ANECDOTES, FACTS, EXTRACTS, ILLUSTRATIONS, QUERIES, SUGGESTIONS, &c.

RELATING TO THE

*History or the Art of Medicine, and the Auxiliary Sciences.*

Quicquid agunt medici, nostri farrago libelli.

### *Anatomico-Physiological Examination of the Medicinal Leech;* by Henry Lebrecht Kurzmann, M.D. Physician to his Majesty the King of Prussia, &c.

*(Concluded from p. 403.)*

THE author had several opportunities to observe the leech while in the act of copulation. They had their tails fixed by suction to the sides of the glass jar, and hung downwards, their bellies twisted around each other. One of them had fastened itself with its mouth to the other, whose head was hanging down. On the bodies being turned a little aside, in the genital regions, the penis of the one was plainly seen to pass, in a spiral form, round that of the other, and then each to enter into the opening of the opposite uterus. Propagation could not, however, be observed; both dying soon after. This act may be still more plainly observed in the horse-leech. Whether the leech belongs to the oviparous or viviparous, cannot be decided with any certainty: Dr. K., however, is in favour of the latter opinion; as, in the glass jars in which they were kept, he perceived, at different seasons, a number of little ones, which always made their appearance so suddenly, that the day before not the least traces were found; and, besides, the water had been frequently renewed, so that their development from eggs could not be supposed.

On each side of the belly fifteen particles are situated in rows, one behind the other; the first close behind the œsophagus, and the last not far from the tail: partly in the same direction with the round substances belonging to the testicles, and partly outwards. Each of these apparently-glandular particles consists of a single closed cylinder, which is finely winding and partly bending itself, whilst the other end is attached in the front and on the sides to the skin of the belly. In young leeches, each of them has scarce the length of a line, is of a white colour, perfectly cartilaginous and smooth; sometimes also covered with a membrane; and erects itself with considerable elasticity, on being irritated with the point of a knife. In old leeches it appears reddish, softer, serpentine, and shows but a trifling erection. The function of this organ is as yet unknown.

The organs of respiration of the leech, or the tracheæ, are flat, round, skinny, vesicles, situated beneath the intestinal canal, sixte n on each side, lying, at equal distances from each other, between the above-described particles; having somewhat more than a line in diameter, and consisting of a fine, white skin, externally, and a somewhat stouter one internally. Each vesicle is overspread by a close vascular texture, which, though the vesicles themselves stand in no connexion among themselves, is connected with those of the others. The exterior openings appear, on the belly-side, as small membranous spots. The vesicles mostly contain a proportionable quantity of a white fluid, which is miscible with water. Mercury introduced into these vesicles, neither proceeding, nor being capable of being forced back, through the external opening, it is supposed that these openings are provided with valves. As the leech, to all appearance, respires in the same manner as fishes do, and of course water enters the vesicles, where it remains till the air contained in it is decomposed, it is very probable, that the leech then opens these valves to let the water run out. The respiratory organs, therefore, deserve rather the appellation of gill-bags, than that of tracheæ.

The vascular system of the leech does not take its origin from one central point, but consists of three apparently separate vessels, one running along the back, and two on each side; all of them of equal strength, and consisting of a very thin, transparent membrane. If an hungry leech, void of blood, is killed by means of alcohol, their course and ramifications become visible, the reddish fluid contained in them congealing into a wax-like mass by this process; and, on opening the moderately-extended leech some hours after, the vascular system, and its branches, are seen as if injected with wax. The vessel on the back begins directly behind the teeth, runs along the back part of the œsophagus, the intestinal canal, and the rectum, to the anus, closely adhering everywhere, to an equal degree, with the parts lying underneath; and small vessels, answering to the divisions of the stomach, are branching off on both sides.

The two side-vessels also run on both sides in a straight direction from the fore-part backwards, on the outside and along the gill-bags, and on the fore-part in three or five thin branches, that lose themselves in the skin, and anastomize with those of the opposite side. Though no fluid can be propelled from the side-vessels into that of the back, Dr. K. is nevertheless of opinion, that there is a connexion betwixt them, but that, on account of the fineness of the vessels, it cannot be ocularly demonstrated. In all these three vessels a pulsation is observed, though in that of the back it is less distinct, on account of its closer consolidation with the adjoining part: ten or thirteen of these pulsations may be counted in a minute. The manner of the circulation may be best observed in the common leech (hirudo vulgaris), in which, on account of its transparency, the interior parts can be seen in a bright sunshine. The circulation does not take place from one end to the other, but from side to side. When one of the side-vessels is distended with blood, that on the opposite side is quite empty; on the former's contracting itself, the round particles of that side fill themselves, then the vessel on the back, the parts of generation, the round particles of the opposite side, and lastly, the other side-vessel; which replenishes itself, till all the other parts from whence the blood proceeded are void: from hence the blood now returns in the same manner to the opposite side. This reciprocal voiding and filling proceeds very slowly, ten or twelve times in a minute at the utmost; and, under the want of influence of the open air, or in stagnant water, still slower; and immediately ceases awhile, on the leech being squeezed or otherwise irritated.

Along the belly, just in the middle towards the back, and under the stomach, coeca, and rectum, runs the principal nerve of the leech, from the head down to the foot. It constitutes a black string, everywhere of the same breadth, on which white spots, constituting the ganglia, are observable, standing at equal distances from each other, making the string a little broader. The sheath of this nerve consists of a simple, firm, membrane, the colour of which appears to be black; but is seen to be brownish-yellow, closely covered with black streaks, when inspected through the glass. These streaks disappear some days after death, and the sheath assumes a whitish-grey colour. Over the ganglion it is, on account of its greater distension in breadth, more closely expanded, and the former appears white through it. On cutting the sheath open, the ganglion presses forward; and, with some caution, the sheath may be stripped from the nerve. The proper nerve is as strong as an ordinary human hair, of a whitish-yellow colour, almost as firm as a sinew, and very elastic; so that it always preserves a straight line, even when the animal contracts itself to half its usual length; but, on its shortening itself still further, it forms slight serpentine windings towards the sides. It may easily be divided in threads, but the ganglion forms an indivisible whole. The nerve in general is rather dry than moist, has twenty-three ganglia, and from these alone are branches issuing forth. The first ganglion, situated over the œsophagus, is small and roundish, sending forth two branches to both sides of the upper-lip, perhaps to what is called the eyes; and two stouter ones backwards, one of which bends to the right, and the other to the left side, of the fore-part of the œsophagus, round that part situated close under the rows of teeth, and then runs below to the belly-side. Here both branches join again in the second ganglion, which is larger, and of a triangular shape. The third ganglion has its situation immediately behind the former, and sends forth on each side a pretty strong branch toward the œsophagus. From the fourth to the nineteenth ganglion, they stand at almost an equal distance from each other, each of them sending forth two branches forward, and two stouter ones backward, in the form of an X; the former passing into the skin of the belly, and the latter into the stomach.

The back branches of the seventh ganglion pass to the testicles. The nervous chord, passing on from this ganglion backwards, bends over the spermatic chord of the right testicle, but directly after joins again the muscular coat. The chord passing from the eighth to the ninth ganglion, divides itself directly after into two branches, surrounding the uterus, without sending to it any branches. From the nineteenth ganglion onwards, they approach each other more and more, so that the twenty-third almost stands in contact with the twenty-second.

The nervous sensibility stands in the leech on a very low scale; the nerve shewing no mark of it, either on being touched with dilated acids, pricked with pins, or touched with a hot needle. Dr. K. cut a leech, fastened at both ends, in such a manner that a connexion existed only between the two halves by means of the nerve, which was uninjured, but deprived of its sheath: the halves directly contracted themselves towards their fastened points, whereby the nerve, and three of its ganglia, were exposed to view. A weak galvanic irritation did, likewise, not produce any alteration. A pile of twenty pair of plates being brought into contact, at both poles, with the nerve and back, or nerve and belly, or either end, produced a slight contracting motion; which became stronger on touching the nerve, and the base of the wound. The touching of the nerve and upper-lip produced a strong retraction of the head; that of the nerve and tail, a strong retraction of the latter; that of the two divided halves, either on the back or belly, strong motions; and, lastly, that of the upper-lip and tail, the most sudden contraction of both halves took place. A leech, prepared in the above-mentioned manner, of which a piece of the muscular coat of the head was deprived of its epidermis, being touched at the bare place and the nerve, or tail, exhibited moderate contractions; stronger ones, on the bare place being touched along with the skin; and still stronger ones, on touching the bare place and upper-lip. A pile of ten pair of plates, did not produce any strong effect upon a living, unprepared leech. One of twenty pair had not any effect, on touching the body with both poles; touching the body and the upper-lip, produced a sudden removal of the same; of the body and foot, a quick removal of the latter; of the upper-lip and the foot, a remarkable quick contraction, and a vomiting of the blood it contained.

To learn which is the immediate influence of the nerve upon the leech, the nerve was taken out, in its whole length, without any of the other viscera being injured, and the leech replaced into the water; when it died in a few hours after. In another leech, a piece of the nerve, with two ganglia, towards the foot, was cut out; in consequence of which, the lower, and below the cut, was paralyzed. The foot gradually recovered some strength, but not its former degree; and the leech died within a few weeks. The leech shews the highest degree of sensibility at the head and foot; in which places the faculty of feeling, the only sense it is possessed of, appears.

The trifling degree of susceptibility of the nervous system of the leech, lets us suppose, that, in the same manner as we perceive a scale in the gradual development of the animal creation, a similar one must be discoverable with respect to the susceptibility for external influences; and a discovery like this would certainly be of great importance for natural history.

The nervous system seems also to act a part in the movement of the leech. The high degree of elasticity of the nerve, in a living condition, whereby it approaches to the nature of a tendon; its being enclosed in a firm, tendinous canal; and the chief spreading of its branches towards the belly; adapt it particularly for it in contractions: and it appears, that all the movements of the leech, notwithstanding its muscles being of equal situation and condition throughout the whole circumference of its body, always determine themselves towards the belly or the sides, according to the situation of the nervous chord and its branches. In the strongest contraction of the leech, a hollow is perceivable along the belly, according to the situation of the nerve, which can only be produced by the contraction of the muscles towards this point; which tendency, as the muscular parts themselves do not go thither, is only to be ascribed to the nerve. Also, in endeavouring to extend a contracted leech, the force of contraction is found to be very strong towards the belly; and, on the contrary, but trifling towards the back.

The vital power of the leech is exceedingly great. On cutting it across, both halves live for weeks, and even months, with an equal vivacity. The skin of the wounds turn inwards, whereby they become smaller, and sometimes actually cicatrize; but, if cut lengthways, both parts die within a few hours. The leeches live in the water, though confined for several years. They live for several days, if put in oil. Alcohol kills them in a few minutes; hot water, instantly; and rose-water, in a few hours. Frost kills them, though the leeches themselves were not frozen, but soft. Its sensibility, by which it is said to predict the change of weather, Dr. K. found not verified. The abode of the leech is in fish-ponds, swamps, ditches, and, in general, stagnant waters; but it never, except by chance, is found in running water. They are caught, in warm sunny days, in calms, or a gentle southerly breeze, when they come up to the surface of the water, on stirring up the swamps, or moving the aquatic plants to which they fasten themselves. Rain-water serves best to preserve them in. A temperate place, which is not exposed to the sun, must be chosen for the stand of the jars containing them. It is not necessary to change the water very often; on the contrary, the change of temperature, unavoidable on those occasions, seems rather to be the cause of their dying sooner. Dr. K. kept leeches alive longer than three years, without ever giving them fresh water during the whole period; except when, on its being too much evaporated, he added some fresh to it: nay, he even has purposely left the dead leeches to putrify in the jar, without its seeming to be detrimental to the living ones.

*Some Observations on the Opinions of the Ancients respecting Contagion.* By G. D. Yeats, M.D. Fellow of the Royal College of Physicians, &c. (From the *Journal of the Royal Institution,* No. xiii.)

An opinion having been promulgated, that the antients disbelieved in the doctrine that fevers were contagious,—that is, that the disease was propagated from one individual to another by contact,—it appeared to me a matter, at least, of curious, if not of useful, research, to enquire how far this opinion was founded in truth. They who will take the trouble to turn over the pages of the ancient historians and poets, will soon find that the description of fevers, both by medical and historic writers, clearly shews that it was the generally-received opinion, that human bodies conveyed to each other febrile infection of a highly malignant nature; and further, it is stated, that diseases were propagated by contagion and infection from brutes to the human race. It would be a matter of grammatical hypercriticism to give the etymology of the word contagion, which, of itself, as so closely connected with its Latin derivation, is sufficient to shew what was the idea entertained of the mode by which some diseases were conveyed from one individual to another. It will not be necessary to look into histories more early than that of Thucydides, although it is related that, after the destruction of Troy, a pestilential disease raged in Greece and the neighbouring countries of Asia; and Herodotus attributes it to the miseries consequent to, and connected with, the Trojan war.

In the second year of the Peloponnesian war, which scourged Greece for twenty-seven years, and which commenced about four hundred years before the Christian æra, a raging pestilence broke out in Athens. An invading army of sixty thousand men covered the beautiful plains of Attica, and compelled thousands of the inhabitants to seek protection within the walls of the already populous and crowded cities; thus generating and increasing, by a pollution of the air in confined habitations, pestilential disease: accordingly, as Thucydides says, ἡ νόσος επενείματο δὲ Ἀθήνας μὲν μάλιςα, ἔπειτα δε και των ἄλλων χωρίων τὰ πολυ ανθρωπότατα.—*Thucydid. Hist.* lib. ii. p. 134. Francofurti, 1594.

"This pestilent disease raged chiefly at Athens, and also in other places where the inhabitants were the most crowded. Diodorus Siculus, in his account of the same pestilence, declares the opinion that the disease arose in consequence of the unusual crowded state of Athens—οἱ δ'Α'θηναῖοι πάρατάξασθαι μεν οὐκ ετολμων, συνεχόμενοι δ'ἐντὸς των τειχων, ἐνέπεσον εἰς λοιμικὴν περίςασιν. πὸλλȣ γαρ πλήθους καὶ παντοδαπου συνέρρυηκοτος εἰς τὴν πόλιν, διὰ την ςενοχωρίαν εὐλόγως εἰς νόσους ἔπιπτον, ελκοντες ἀερα διεφθαρμενον.—Lib. xii. p. 101. "The Athenians, not daring to meet the Peloponnesians in open battle on the plain, remained cooped-up within their walls, and caused pestilential effluvia; for, great multitudes of people from all quarters congregating in the city, very readily generated disease by breathing a corrupted air." The eloquent and animated description which Thucydides gives of the symptoms, clearly describes a fever of the most violent kind. It was attended with such violent thirst and evolution of animal heat, that the miserable sufferers threw themselves into the sea, into ponds, and even into the wells, to quench their thirst and raging heat. The art of the physicians not only was of no avail, but they themselves, and all who approached the sick, were cut off by the contagion:—ἀλλ' ἀυτοὶ μάλιςα ἔθνησκον ὅσω καὶ μάλιςα προσῄεσαν.—P. 129. Such was the dread created by thus catching the contagion, that people were unwilling to attend the sick; there was a mutual fear of visiting each other; and whole families perished in consequence of want of assistance; and they who braved the danger, from a principleof virtuous affection in attending their sick friends, perished in heaps: —καὶ ὅτι ἕτερος ἀφ' ἑτέρε θεραπείας αναπιμπλάμενοι, ὥσπερ τα πρόβατα ἔθνησκον· καὶ τὸν πλεῖςον φθόρον τῦτο ἐνεποίει. εἴτε γὰρ μη θελοιεν δεδιότες ἀλλήλοις προσιέναι, απωλλυντο ἔρημοι, και οἰκιαι πολλαὶ ἐκενώθησαν ἀπορία τε θεραπεύσοντος, εἴτε, προσίοιεν, διεφθείροντο.—P. 132. Thus, then, it appears clearly from the account of Thucydides, that the contagion not only spread from one individual to another, but, what is very remarkable, as shewing the belief of the virulence of the disease caught in this way, he adds, that the greatest part of the mortality was produced by this communication of the contagion:—καὶ τὸν πλεῖςον φθόρον τῦτο ενεποιει; and, in the popular clamour which was raised against Pericles for involving his country in the destructive Peloponnesian war, he was accused, says Plutarch, of giving more violence to the pestilence which raged at Athens, by keeping the people cooped-up like herds of cattle, to be infected with contagion from one another:—ἀλλ' ἐων ὥσπερ βοσκήματα καθειργμένοις, ἀναπίμπλασθαι φθορᾶς απ' ἀλλήλων.—Plutarch, vita Periclis.

Aristotle, the son of a physician, has, in one or two of his problems, proposed questions for reasons why diseases should be propagated from a diseased person to a sound one who approaches him. So prevalent was the opinion of the contagious nature of pestilential diseases, that he puts it down as a problem:—Διὰ τι ποτε ὁ λοιμὸς μόνη τῶν νοσων μάλιςα τὺς πλησιάζοντας τοῖς θεραπευομένοις προσαναπίμπλησιν; ἢ ὅτι μόνη των νόσων κοινῆ ἐςιν ἅπασιν. ὥςε διὰ τῦτο πᾶσιν επιφέρει τον λοιμὸν, ὅσοι φαυλως ἔχοντες προϋπάρχυσι, καὶ λὰρ διὰ τὸ υπεκκαυμα τῆς νοσε της παρὰ τῶν θεραπευομενων γεινομένης, ταχέως υπὸ τῦ Πράγματος ἁλίσκονται.—Sect. 1, Prob. vii.

"From what cause does it happen that the plague alone, of all diseases, especially infects those who approach the persons labouring under it? Whether is it that, of all diseases, mankind are more susceptible of it? Therefore, on this account, the plague attacks all who, being of a bad habit, are first seized with it; for, a fomes of the disease being generated in those labouring under it, others are quickly infected with it."

No doubt can possibly be entertained here of the opinion respecting the contagious nature of plague: on the contrary, the opinion is so established and believed, that it is asked why it should be so? It is also not a little curious, that Aristotle should state, that the plague first commences in those who are of a bad habit of body; or, to speak in modern language, he conceived a predisposition of the constitution rendered the body more susceptible. The constitution, being thus impregnated with disease, generated a fomes, which readily communicated the contagion to another. I take υπεκκαυμα, to be very expressive in this way. Thus we have the complete modern doctrine explicit and clear in a single problem of Aristotle,—the susceptible predisposition of the body in taking infection, the generation of a fomes, or infectious principle, readily communicating the disease to others by contact. In the eighth problem of the seventh section, are some more explanations on this point; in which he observes, all are easily affected with such diseases as arise from a corrupted source, such as pestilences; for they who approach such are immediately infected:— ὁ δε πλησιάζων τοιῦτον ἀνάπτει.

In various parts of Diodorus's history, we find accounts of pestilential diseases as they occurred in different parts of the world, particularly among multitudes of people collected together for the purposes of war. A contagious pestilence broke out at Carthage at the time it was invaded by Dionysius, the tyrant of Syracuse. Diodorus, in his account of this pestilence, (the symptoms of which he has described,) particularly points out the infection and fatality produced by approaching the sick:—μετα δε ταῦτα διὰ τε τὸ πληθος των νεκρων και το τοὺς νοσοκομῦντας ὑπὸ τῦ νόσῦ διαρπάζεσθαι, ουδεὶς ετολμα προσιέναι τοῖς κάμνοισι.

"As the mortality caused by the disease was great, and as the attendants upon the sick were cut off by it, no one dared to approach the infected."—Again:

Καὶ γὰρ οἱ τοῖς κάμνοισι παρεδρεύοντες ἐνέπιπτον εἰς τόν νόσον παντ ες ὥςε δεινὴν εἶναι τὴν συμφορὰν των ἄρρωςούντων, μηδενὸς θέλοντος ὑπηρετεῖν τοῖς ἀτυχουσιν, ού γαρ οἱ μηδὲν προσήκοντες ἀλληλους ἐγκατέλειπον, αλλ αδελφοὶ μὲν αδελφοῖς, φίλοι δὲ τῦς συνήθεις ἠναlκάζοντο προίεσθαι διὰ τὸν ὑπερ αὑτων φόϐον.

"For all took the disease who had close communication with the sick, so that wretched indeed was the condition of those who were diseased, every one being unwilling to assist them; for, not only they who were not bound by any tie of relationship deserted each other, but brothers and friends were compelled to neglect their nearest relatives and companions, on account of their dread of the contagion."

In various parts of the history of the Romans, by the Halicarnassian historian, we find accounts of pestilential fevers which spread havoc and destruction around; and we not only can discover that these fevers were infectious, by the manner in which they spread, but Dionysius expressly tells us that they who touched, or lived with, persons so diseased, became infected. In the 451st year before the Christian æra, and about 300 after the building of the city, a contagious fever broke out in Rome:— Δοιμικὴ νόσος εἰς τὴν Ρώμην κατέσκηψε, μεγίςη των εκ τῦ προτέρῦ χρόνῦ μνημονευομένων υφ' ης οἱ μεν θεράποντες ὀλίγῦ εδεησαν ἅπαντες ἀπολεσθαι, των δὲ ἄλλων πολιτῶν ἀμφὶ τῦς ἡμίσεις μαλιςα διεφθάρησαν, ῦτε των ἰατρὼν ἀρκῦντων ἔτι βοηθεῖν τοῖς καμάτοις, ῦτε οἰκειών ἢ φίλων τα ναγκᾶια ὑπηρετῦντων οἱ χαρ επικῦρεῖν τᾶις ἑτέρων βῦλόμενοι νόσοις, απτόμενοι τε καματηρων σομάτων, και συνδιαιτώμενοι τὰς αυτὰς ἐκείνοίς νόσῦς μετελαμ-

Caror.—*Dionysii Halicarnassensis Historia.* Oxoniæ, 1704. p. 645.

"The most pestilential disease ever remembered brought destruction upon the city, by which almost all those affording assistance were cut off, and nearly one-half of the other citizens were destroyed; neither were the physicians able to attend effectually the sick, nor the friends and domestics to administer the necessaries; for they who willingly attended others, by touching their diseased bodies, or dwelling with them, were seized with the same malady."

Here we have both contagion and infection clearly stated: the former communicated by touching the diseased bodies, the latter giving disease to those who came within the concentrated sphere of its action. The miserable condition of the city, during the ravages of this pestilence, is pathetically described by Dionysius. The stench from the unburied dead bodies, which were cast into the sewers, on the highways, and into the river, and again thrown by the tide upon its banks, contributed to maintain the disease, by spreading the infection; for such was the desolation, that whole houses were deprived of their inhabitants by death. The contagion was carried into the country, where numbers perished; and the Æqui, the Volsci, and the Sabines, enemies of Rome, desirous of taking advantage of the distresses of the city, by invading it, received the infection, and carried destruction into their own habitations. The yeomanry of the country were either destroyed or paralysed by the febrile infection; the ground remained untilled, and famine was added to pestilence.

Interspersed in various parts of Livy will be found histories of pestilential fevers, the infection of which, it is expressly stated, was spread by contact. About the year 389, A.U.C. a fever of this kind broke out in Rome.

"Grave tempus et fortè annum pestilens erat," says Livy, "urbi agrisque nec hominibus magis quam pecori; et auxere vim morbi terrores populationis, pecoribus agrestibusque in urbem acceptis. Ea colluvies mistorum omnis generis animantium et odore insolito urbanos et agrestem confertim in arcta tecta æstu ac vigiliis angebat, ministeriaque invicem et contagio ipsa vulgabant morbos."—Lib. 3. c. vi. p. 47.

Again, in the account which Livy gives of the fever which broke out among the soldiers during the siege of Syracuse, a siege ever remarkable by the death of Archimedes, it is clearly stated that contact of the sick propagated the disease.

"Accessit et pestilentia, commune malum, quod facile utrorumque animos averteret a belli consiliis, nam tempore autumni et locis naturâ gravibus, multo tamen magis extra urbem quam in urbe intoleranda vis æstûs per utraque castra omnium fermè corpora movit, et primo temporis ac loci vitio et ægri erant et moriebantur, postea curatio ipsa et contactus ægrorum vulgabat morbos; ut aut neglecti desertique, qui incidissent, moriuntur aut assidentes curantesque eâdem vi morbi repletos secum traherent."—Lib. xxv. c. 26. Carthaginians, Romans, Sicilians, all fell victims to the disease.

So prevalent, indeed, was the opinion of the contagious nature of pestilential fever or plague, that we find in medical writings, when the authors wish to represent the infectious nature of disease, they compare it to the plague. Thus Aretæus, one of our best and most accurate professional writers, in giving an account of the nature of Elephantiasis, and wishing strongly to impress on his readers the idea of its highly-contagious nature, says,—ἀτερπὲς μεν και φοϐερὸν, θηρίȣ γαρ ἰδέν, δεος δὲ ζυμϐιȣντε και ξυνδιαιτᾶσθαι, ȣ μεῖον ἢ λοιμῶ· αναπνοῆς γαρ ες μετάδοσιν, ῥηιδίη βαφή.—Θεραπεια Ελεφαντος.

"It is a terrible and unsightly disease, putting on the appearance of the beast: there is great danger, too, in taking food with one so diseased, as much as with one afflicted with the plague; for the infection is readily caught by a reciprocity in respiration."

Galen, too, in his first book, chapter iii. *De Febribus*, not only alludes to, but directly asserts, the contagious nature of the plague; for he says, in strong language, there are none, possessing any understanding, who do not know that a pestilential condition of the air will produce fevers; as also that it is very imprudent to have any direct communication with those afflicted with the plague, on account of the danger of catching it, in like manner as in the itch or inflammation of the eyes:—κὰι μεν δὲ κὰι ὅτι λοιμώδοις ἀέρος κατάςις ηνεγκε πυρετον, ȣδὲ τȣ̃το αγνοȣ̃οσιν, οις μέτεςι συνεσεως· ωσπερ γε καὶ ὅτι συνδιατρίϐειν τοῖς λοιμωτοισιν ἐπισφαλές απολαυσαι γαρ κίνδυνος ὥσπερ ψώρας τινός ἢ οφθαλμιας.

It may not be incurious to remark here, that ophthalmia is said to be contagious; and Aristotle asserts the same thing, in the eighth problem of the seventh section. What is the reason, he says, that they who have close intercourse with persons labouring under the itch, or ophthalmia, are seized with it?—Διὰ τί απὸ φθίσεως και ὀφθαλμιάς και ψώρας οι πλησιάζοντες ἁλίσκονται.

The public are well acquainted with the discussion which has taken place respecting the contagion of that species of ophthalmia which so sorely afflicted our army in Egypt.

In the sixth chapter of the fourteenth book of the history of Ammianus Marcellinus, where he describes the vices of the people of Rome, he alludes to a disease of a highly-infectious nature, at a period of time about 353 years after the birth of our Saviour. It appears to me to be almost impossible to say what the disease was; but it is sufficient to state, that the account describes it to be so exceedingly infectious, that the servants sent to enquire after those who were ill, were ordered to undergo purification before they returned home;—"Et quoniam apud eos, ut in capite mundi, morborum acerbitates celsius dominantur; ad quos vel sedandos omnis professio medendi torpescit: excogitatum est adminiculum sospitale, ne quis, amicum perferentem similia, videat: additumque est cautionibus paucis remedium aliud satis validum, ut famulos percontatum missos quemadmodum valeant noti hâc ægritudine conligati, non ante recipiant domi, quam lavacro purgaverint corpus. Ita etiam alienis oculis visa metuitur labes." Here, we have not only the dread of contagion being communicated from one individual to another, but precautions were taken to prevent the infection being carried by the clothes or person of a second individual to a third. Whatever particular condition or nature of disease "labes" may be supposed to mean, it is, nevertheless, perfectly clear, that an infectious principle, communicable through the medium of another, was feared and avoided. Ammianus, however, in the fourth chapter of the ninth book, describing a pestilence which raged at Armida in Mesopotamia, when it was besieged by Sapor king of Persia, A.D. 359, and in giving the different opinions which were held respecting the origin of pestilential diseases, makes the following observations, in which "labes" is evidently used as disease :—" Adfirmant etiam aliqui, terrarum halitu densiore crassatum aera emittendis corporis spiraminibus resistentem, necare nonnullos: quâ causâ animalia præter homines cætera jugiter prona, Homero auctore, et experimentis deinceps multis cum talis incesserit labes, ante novimus interire."

To the facts which have been adduced from medical and historical writers respecting contagion, may be added the descriptions and opinions of the poets. The classical reader will feel a pleasing interest in bringing to his recollection the readings of his earlier days; and will, therefore, be gratified by the perusal of quotations from the writings of those authors whom he has probably often had in his hand. In the beautiful description, from the first eclogue of Virgil, where he characterizes himself under the name of Tityrus, and the Mantuans under that of Melibœus, (who had been spoiled of their lands to enrich the followers of Augustus,) the latter thus addresses him :

> Non insueta graves tentabunt pabula fœtas;
> Nec mala vicini pecoris contagia lædent.

Thus, as on a subject well known, Melibœus congratulates Tityrus that his cattle will not be exposed to the contagion of a neighbouring herd; as, by the interest he had, through Mecænas, with Augustus, he was permitted to retain his Mantuan property, and was not, therefore, obliged to remove his flocks to other and untried pasturage. In the third book of the Georgics, Virgil is still more explicit and clear respecting the contagious nature of disease in cattle; for he describes the symptoms of one becoming ill, and desires it may be immediately attended to:

> Prius quam
> Dira per incautum serpant contagia vulgus.

The other Roman poets of nearly the same period, are equally descriptive and explicit in their accounts of pestilential diseases, as propagated by contagion, as we read in the pages of Lucretius, Ovid, Lucan, and Silius Italicus. I cannot avoid the pleasure of quoting, from the two former at least, their highly-poetical descriptions. Lucretius, in his beautiful account of pestilential fever, unquestionably taken from the historical statement of Thucydides, evidently describes its infectious nature. Its malignancy and rapid propagation from one individual to another, by contagion, are unequivocally stated;

Quippe etenim nullo cessabant tempore apisci
Ex aliis alios avidi contagia morbi.
Lanigeras tanquam pecudes et bucera sêcla;
Nam quicunque suos fugitabant visere ad ægros,
Vitai nimium cupidi mortisque timentes
Pœnibat paullo post turpi morte malâque,
Desertos, opis expertis incuria mactans,
Qui fuerant autem præsto, contagibus ibant.

Ovid, in the seventh book of his Metamorphoses, gives an animated and highly-poetical account of the plague which raged in the island of Ægina; and we find the symptoms and calamitous circumstances attending the disease, similar to those as described by the masterly pen of Thucydides. We must therefore conclude, that it is either a fiction of the poet's, as a copyist, or an account of a disease, similar to the Athenian pestilence, as it occurred in an island of the Grecian Archipelago. However that may be, the idea of the contagious nature of the pestilence is determined and unequivocal, clearly shewing, that the poet is stating an opinion generally received in his, and the preceding, times. After describing the calamitous havoc caused by the spreading of the disease, the fields and roads being strewed with the bodies of the dead, the air corrupted with the effluvia of the putrefaction, he adds, the contagion is thus spread far and wide:

* dilapsa liquescunt;
Afflatuque nocent et agunt contagia latè.

Human aid is of no avail; for the faithful attendant, in his solicitous care of the sick, by a near approach, catches the contagion, and is thereby hastily hurried to his grave:

Nec moderator adest: inque ipsos sæva medentes
Erumpit clades, obsuntque autoribus artes;
Quo proprior quisque est, servitque fidelius ægro
In partem lethi citius venit.

Instead, then, of having any doubts on the opinions of the ancients, respecting the propagation of disease by contagion and infection, we have ample proof, from the writings of their philosophers, physicians, and poets, not only of the existence of such an opinion, but of precautions taken to prevent the spreading of the infection. In the reading, however, which I have gone through, I do not recollect to have met with a passage describing any strictly precautionary means, except in Ammianus Marcellinus. I am, nevertheless, inclined to believe that, by a deeper perusal of the ancient Greek and Roman authors, we should find that methods were adopted for preventing the communication of contagious miasma; not, however, with that philosophical accuracy and success which the improvements in modern science have produced. Many works, besides those I have read, remain to be examined, as well as a more critical perusal of them; but what I did read, was so much to the point, and so satisfactory, that it was unnecessary to proceed further.

* Scilicet Corpora.

